# Automatic detection of moral information: evidence from visual mismatch response (vMMR)

**DOI:** 10.1186/s40359-026-04948-x

**Published:** 2026-06-10

**Authors:** Bo Sun, Shasha Guo, Zenghua Xing, Xixi Gu

**Affiliations:** 1 Institute of Psychology and Behavior, Henan University, Kaifeng, China; 2 School of Sociology, China University of Political Science and Law, Beijing, China

**Keywords:** Moral judgment, Moral intuition, Visual mismatch response, Event-related potentials (ERPs)

## Abstract

**Background:**

A theoretical debate persists regarding whether moral judgment is primarily driven by moral intuition or moral reasoning. Furthermore, it remains unclear whether moral information is processed automatically, despite the social intuitionist model’s assertion that moral judgments arise from rapid, intuitive processes.

**Method:**

To investigate the automaticity of moral judgment, the present study employed the visual mismatch response (vMMR) as an electrophysiological marker. The vMMR is an event-related potential component that indexes the brain’s automatic detection of environmental changes. Using a reversed block design, we presented moral and immoral stimuli while manipulating task relevance.

**Results:**

The results revealed that only immoral stimuli elicited an early vMMR, and its amplitude was unaffected by task relevance. In contrast, both moral and immoral stimuli elicited a late vMMR.

**Conclusions:**

These findings suggest that the automatic detection of moral information, consistent with the notion of moral intuition, occurs during the early stage of moral judgment and operates as a highly automatic and negatively biased process. Moreover, the presence of both early and late vMMR indicates that moral judgment involves multi-stage automatic processing, encompassing both rapid deviance detection and subsequent, more elaborate automatic analysis.

## Background

In everyday life, individuals frequently evaluate the morality of actions, prompting theoretical debate about whether moral judgment primarily arises from moral intuition or moral reasoning. Moral intuition can be defined as a fast, automatic, and usually unconscious, affect-laden psychological process that enables an individual to produce a moral judgment almost instantaneously when faced with a moral situation [1, 2]. Within moral psychology, different perspectives exist on this issue. One view holds that moral judgment results from moral reasoning, where deliberate cognitive processes determine subsequent moral evaluations [3]. In contrast, the social-intuitionist model posits that moral judgment is predominantly shaped by rapid, automatic intuitions, with moral reasoning serving merely as a post-hoc justification [1]. Notably, the cognitive-intuitionist model adopts an integrated stance, suggesting that both moral intuition and moral reasoning jointly guide moral judgment [4].

Using implicit rather than explicit moral tasks provides a more effective means of investigating the early processing of moral information. Prior research has mainly focused on examining neural activity during moral judgment [2, 5, 6]. For instance, Yoder and Decety [6] employed explicit moral tasks to explore the spatiotemporal neural dynamics of moral judgment. Their findings revealed that early ERP components such as N1 and N2 differentiate moral valence, while the late positive potential reflects cognitive reappraisal of moral content. However, these studies are limited by their reliance on explicit tasks, in which participants are directly instructed to evaluate the moral content of stimuli. This approach ensures that participants are fully aware of the moral nature of the stimuli, thereby engaging processes more aligned with moral reasoning [7]. Moreover, explicit tasks allow participants to deliberately control their responses, which may obscure their true attitudes [8]. Consequently, explicit moral tasks are not well-suited to capturing moral intuition and offer limited insight into automatic moral processing. In contrast, implicit moral evaluation—characterized by passive attention and spontaneous reactions to moral information—better reflects fast moral processing [7]. Unlike explicit tasks, in implicit moral tasks participants are not informed that the stimuli contain moral content nor asked to make moral judgments. Instead, they are explicitly instructed via task directions to attend and respond to morally irrelevant features of the stimuli (e.g., gender, age, or location), while the moral dimension remains entirely task‑irrelevant.

The degree to which moral information processing operates automatically warrants further investigation. According to the social-intuitionist model, moral judgments are primarily driven by fast and automatic moral intuitions [1]. Supporting this view, Tao et al. [9] employed an implicit moral task and observed that moral information processing occurs rapidly, as indexed by the N2 component. In their study, participants were asked to view morally relevant pictures and determine whether the pictures contained animals or children. Critically, while the moral content of the pictures was not mentioned and was thus task‑irrelevant, the pictures themselves served as the target stimuli that participants had to actively attend to and categorize. In other words, the stimuli carrying moral information were task‑relevant (participants had to process the pictures to perform the task), even though the moral dimension was task‑irrelevant. This design leaves open the possibility that participants’ attention was drawn to the pictures per se, allowing moral features to be consciously processed, and thus the N2 effects may not reflect fully automatic processing under non‑attentive conditions. Second, low-level perceptual differences between moral and immoral stimuli (e.g., the presence of physical harm cues) may have confounded the results: the observed ERP differences could be driven by these low-level variations rather than by moral content itself.

To overcome these limitations, the present study employs the visual mismatch response (vMMR) as a neural indicator. Moreover, we directly address the issue of attention by introducing a task‑relevant but morally‑irrelevant condition. In our paradigm, morally relevant pictures are presented as background stimuli while participants either perform a demanding fixation task (task‑irrelevant condition, where the pictures are entirely unattended) or are required to attend to the pictures to detect rare neutral targets (task‑relevant condition, where the pictures themselves are attended but their moral meaning remains completely task‑irrelevant). This manipulation allows us to disentangle attention to the stimulus carrier from attention directed at its moral dimension, providing a more rigorous test of the automaticity of moral information processing.

vMMR is an event-related potential component that reflects the brain’s automatic detection of changes in the visual environment [10]. It is typically elicited in an oddball paradigm, in which a regularity established by frequently presented standard stimuli is occasionally interrupted by a deviant stimulus [10]. The vMMR is characterized by amplitude differences between deviant and standard stimuli at posterior electrodes, occurring approximately 150–400 ms after stimulus onset [11]. Because vMMR indexes pre-attentive regularity extraction and prediction violation, it serves as a sensitive tool for investigating whether the brain automatically detects deviations in complex information, even when that information is task-irrelevant. For example, Stefanics et al. [12] used an oddball paradigm to examine automatic processing of unattended facial emotions and found that regular presentations of happy faces, when occasionally interrupted by fearful faces, elicited vMMR. Notably, previous studies have reported inconsistent findings regarding the polarity of vMMR, with some observing visual mismatch negativity [12–14] and others reporting visual mismatch positivity [15–17]. Therefore, Sulykos and Czigler [17] recommended using the more neutral term vMMR.

Using vMMR as an indicator offers a novel approach to investigating implicit moral judgment. Predictive coding theory posits that the brain integrates prior experiences to infer the causes of perceptual input, forming predictions about the external environment [18]. When deviant stimuli violate these predictions, vMMR is elicited. This process involves the automatic extraction of regularities from repeated stimuli and the generation of predictions, aligning with implicit category formation [10]. Therefore, vMMR is well-suited for investigating whether frequently encountered moral information can be automatically detected and categorized without conscious awareness. It also enables examination of whether prediction error signals are generated when moral information deviates from established regularities.

The present study uses vMMR as an indicator to explore the automaticity of moral judgment. Previous vMMR research has demonstrated that individuals can automatically detect changes in simple visual features [13, 17, 19] and facial emotion [15, 20, 21] under non-attentive conditions. However, it remains unknown whether moral information can be processed automatically in a similar manner. The current study aims to address this gap. To ensure attention was engaged elsewhere, participants performed a task requiring discrimination of infrequent changes in a central fixation cross. Meanwhile, morally relevant pictures presented in the background were entirely task-irrelevant, and participants were not asked to evaluate their moral content. This design provides a more rigorous test of the automaticity of moral information processing. Additionally, to control for low-level perceptual confounds, the same stimuli served as both deviants and standards across different experimental sequences.

The relationship between attention and the automatic processing of moral information warrants further clarification. The automaticity of moral intuition may stem in part from the inherent salience of moral content, whereby such information spontaneously captures attention due to its high social relevance and distinct perceptual features [22–26]. Howevter, a central question remains: does the automaticity of moral intuition depend on the active engagement of attentional resources? To address this, the present study manipulates task relevance to disentangle attention to the stimulus itself from attention directed to its moral dimension, thereby examining how attention influences the automatic processing of moral information. Specifically, we introduced a “task-relevant but morally irrelevant” condition. Under this condition, participants were required to actively attend to the stimulus pictures to make judgments about neutral targets, without being instructed to process their moral meaning. If moral information elicits similar early vMMR responses under both task-relevant and task-irrelevant conditions, this would provide evidence that the processing of moral information is highly automatic and not modulated by task-directed attention. Furthermore, this design helps investigate, within the predictive coding framework, the role of attention in modulating the precision of prediction error signals [27].

Whether moral information can be processed automatically remains an open question. Although some studies have shown that moral information can be processed rapidly, as reflected by early ERP components such as N1 and N2 [2, 9, 28], most of the supporting evidence comes from studies using explicit moral evaluations or task-relevant stimuli. These approaches likely involve conscious processing and attentional modulation, thereby complicating the interpretation of automaticity [7]. For example, even in implicit paradigms, participants may still notice moral content if it is part of the task-relevant stimulus set [9]. Furthermore, comparisons between moral and immoral stimuli are often confounded by low-level perceptual differences. To address these issues, we use vMMR as a methodological tool. By observing vMMR in response to moral deviants under strictly unattended conditions, we can infer that the brain automatically detects deviations in moral content—a process consistent with the notion of rapid moral intuition. This approach, with perceptual confounds controlled, allows for a more rigorous test of the automaticity of moral information processing. By further manipulating task relevance, we examine whether attention modulates this early detection stage.

The hypotheses of this study are as follows. First, based on the social-intuitionist model’s claim that moral intuition is fast and automatic [1], we hypothesize that if the brain can automatically detect deviations in moral content, this should be reflected in a vMMR. Second, we anticipate that the automatic detection of moral information will elicit both early and late vMMR, potentially reflecting distinct stages of automatic analysis. Given that moral information is task-irrelevant in our design, any observed late vMMR would more parsimoniously be interpreted as a later, more fine-grained stage of automatic processing rather than as an index of conscious moral reasoning. Finally, we further explore the degree of automaticity of this process by manipulating selective attention to morally relevant stimuli through task relevance. Given that moral intuition is proposed to be highly automatic [1, 4], we expect that the amplitude of the early vMMR will not be modulated by task relevance.

## Materials and methods

### Participants

Twenty-three college students participated in this experiment. Data from two participants were lost due to equipment malfunction, and one participant was excluded due to low accuracy (45%). Finally, the data of 20 participants (14 females, mean age = 24.05, SD = 2.28) were included in the analysis. All participants were right-handed and had normal visual acuity or corrected visual acuity. Also, they had no history of psychiatric disorders. To detect a significant three-way interaction among moral valence (moral vs. immoral), task type (relevant vs. irrelevant), and stimulus type (standard vs. deviant), the required sample size was estimated using G*Power 3.1. With an effect size (ƒ = 0.25), the planned sample size was 16 (α = 0.05, 1–*β* = 0.8). Therefore, the sample size of this study met the requirements. Prior to the experiment, all participants signed an experimental informed consent form. The experiment was approved by the local ethics committee. Participants were recruited through campus advertisements. All participants received monetary compensation for their participation.

### Apparatus

The experiment programs were written using E-Prime 2.0 (Psychology Software Tools, Inc., Pittsburgh, United States). The computer screen has a refresh rate of 60 Hz and a resolution of 1440 × 900 pixels with a viewing distance of 57 cm.

### Stimuli

In the present study, the moral content of the stimuli was restricted to the harm/care domain. This decision was guided by Moral Foundations Theory (MFT), which posits that human moral concerns are built upon several innate and culturally shaped foundations, including care/harm, fairness/cheating, loyalty/betrayal, authority/subversion, sanctity/degradation, and liberty/oppression [29, 30]. Since different moral foundations may engage distinct psychological processes and introduce potential confounds [31], we limited our stimulus set to a single moral domain (harm/care) to enhance experimental control.

**Fig. 1:**
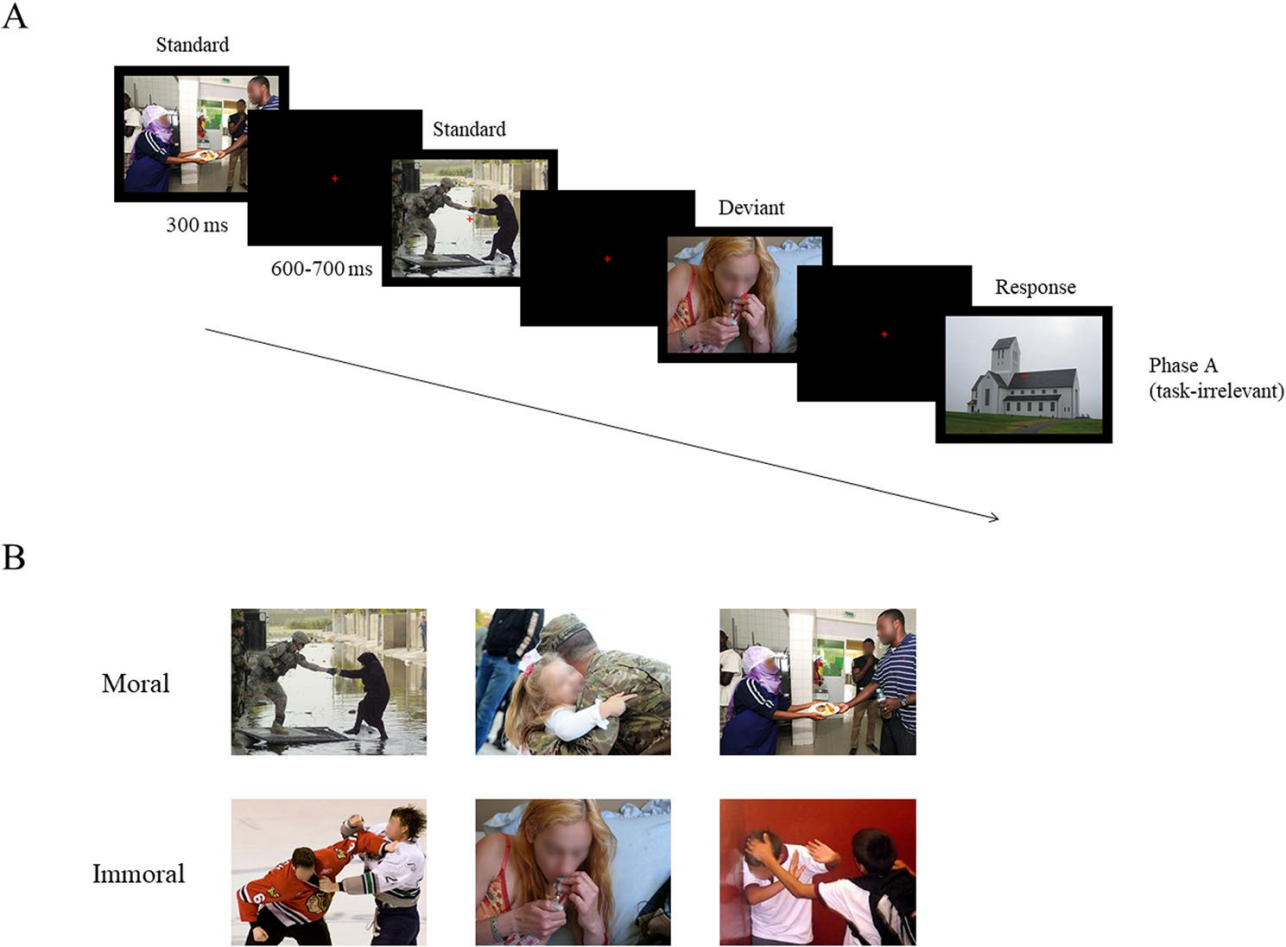
**A** Schematic illustration of the experimental procedure in phase A. Each trial presented morally relevant stimuli for 300 ms. After an interval of 600–700 ms, the next trial followed. As shown in the figure, moral stimuli were standards and immoral stimuli were deviants. In phase A, participants were instructed to detect changes in the central fixation cross (from a cross to a vertical or horizontal red line) and respond by pressing a button. The red line in the figure is used to illustrate the change and is not overlaid on the actual picture stimulus. The procedure in Phase B was similar to Phase A, except that participants were required to detect the appearance of target pictures (a house or a forest). **B** Examples of the moral and immoral stimuli

Furthermore, two researchers selected morally relevant images depicting harm/care scenarios from the Socio-Moral Image Database [32] and ensured that the selected image stimuli had no word information. Several pictures were obtained from the Internet. The dataset consisted of 12 moral pictures, 12 immoral pictures, and 2 neutral pictures (a house and a forest), with each picture having dimensions of 300 × 225 pixels. Examples of the moral and immoral stimuli can be seen in Fig. 1. The ratings of the image materials indicated differences in moral valence and harm between moral and immoral stimuli, with no difference in arousal. The details about the materials ratings are as follows. Prior to the formal experiment, the moral content, emotional arousal, and harm/care dimensions of the pictures were rated on a scale from 1 to 9 by 30 participants (25 females, mean age = 20.70, *SD* = 2.39) (see Table 1). Repeated-measures ANOVA with picture type (moral, immoral, and neutral) was conducted separately for each dimension. The findings from the analysis revealed the following: (1) Moral content: The main effect of picture type was significant [*F*(2, 58) = 338.77, *P* < 0.001, $\:{\eta\:}_{p}^{2}\:$= 0.92], the moral content of moral pictures was higher than that of immoral (*P* < 0.001) and neutral pictures (*P* < 0.001), and the moral content of neutral pictures was higher than that of immoral pictures (*P* < 0.001); (2) Emotional arousal: The main effect of picture type was significant [*F*(2, 58) = 38.36, *P* < 0.001, $\:{\eta\:}_{p}^{2}\:$= 0.57]. There was no difference in emotional arousal between moral and immoral pictures (*P* = 0.103), whereas neutral pictures elicited lower arousal compared to moral (*P* < 0.001) and immoral pictures (*P* < 0.001); (3) Harm: The main effect of picture type was significant [*F*(2, 58) = 316.65, *P* < 0.001, $\:{\eta\:}_{p}^{2}\:$= 0.92]. The harm of moral pictures was lower than that of immoral pictures (*P* < 0.001) and neutral pictures (*P* = 0.01), and the harm of neutral pictures was lower than that of immoral pictures (*P* < 0.001).

**Table 1:** Pre-test ratings of moral content, emotional arousal, and harm of stimuli [*M* (*SE*)]

	Picture type		
	Moral	Immoral	Neutral
Moral	7.98 (0.13)	2.43 (0.15)	6.15 (0.24)
Emotional arousal	5.79 (0.33)	6.32 (0.22)	3.68 (0.35)
Harm	1.41 (0.09)	7.03 (0.17)	2.13 (0.24)

### Procedure

To minimize the influence of low-level stimulus differences, the present study employed a reversed block design [12, 15, 33]. In this design, the same physical stimulus serves as both the deviant and the standard across different sequences, enabling the isolation of the vMMR elicited by identical stimulus parameters. Specifically, in the oddball sequence, moral stimuli served as standards and immoral stimuli as deviants, whereas in the reversed block sequence, immoral stimuli served as standards and moral stimuli as deviants. The order of the two sequences was randomized. The duration and presentation method of the experimental stimuli were adopted from Hu et al. [34].

The experiment comprised two phases (A and B), each consisting of 16 blocks. Within each phase, two sequences—an oddball sequence and a reversed block sequence—were administered, with eight consecutive blocks per sequence. Each block contained 81 trials, with 60 standards, 15 deviants, and 6 trials requiring a button-press response. Deviants were never presented on consecutive trials. On each trial, morally relevant pictures (subtending 9° × 6.2° of visual angle) were displayed at the center of the screen for 300 ms against a gray background, with a variable interstimulus interval ranging from 600 to 700 ms. A central fixation cross (0.4° × 0.4°) remained on screen throughout the experiment. The moral and immoral pictures presented on each trial were pseudorandomly drawn from two sets of 12 pictures each. Neutral pictures depicting houses and forests were presented on trials requiring a response.

In Phase A, participants were instructed to detect changes in the fixation cross, while the morally relevant pictures were task-irrelevant. The fixation occasionally changed to a vertical or horizontal line (0.6°), with each type of change occurring three times per block. Participants were required to press a button (using the 4 or 6 keys on the main keyboard, counterbalanced across participants) as soon as they detected a change. These fixation changes always coincided with the onset of the picture stimuli and lasted for 300 ms.

In Phase B, the moral pictures were task-relevant, though their moral content was irrelevant to the task. Participants were instructed to respond to neutral target pictures (houses and forests) by pressing the corresponding buttons (again, the 4 or 6 keys, counterbalanced across participants), while the fixation remained unchanged. Each block contained three presentations of house pictures and three presentations of forest pictures. The order of Phase A and Phase B was counterbalanced across participants. Following the EEG recording, participants rated the pictures on three dimensions, namely moral content, harm, and emotional arousal, using a 9 point scale.

The manipulation in this study was designed to disentangle attention to the visual stimulus itself from intentional processing of its moral dimension (see Table 2). In the task-irrelevant phase (Phase A), attentional resources were fully engaged by the central fixation task, rendering the moral pictures unattended background stimuli. Here, moral information was processed under unattended conditions. In the task-relevant phase (Phase B), the pictures themselves became the focus of attention, but their moral content remained task-irrelevant. Participants attended to the pictures to detect rare neutral targets, ensuring the stimuli were visually processed, without any instruction to evaluate their moral meaning. This manipulation tests whether moral content is processed automatically and unintentionally when the carrier stimulus is attended or unattended. By comparing these two phases, we can assess whether attention to the visual features modulates the neural response associated with the automatic processing of moral information.

**Table 2:** Overview of the experimental design logic

Phase	Task type	Participants’ task	Role of morally relevant pictures (moral/immoral)
A	Task-irrelevant	Detect changes in fixation cross (cross to vertical/horizontal line) and press button	Presented as background; not attended
B	Task-relevant but morally irrelevant	Detect neutral target pictures (house, forest) and press button	Presented as stimuli; attended to detect targets

### EEG recording and preprocessing

Electroencephalographic (EEG) data were recorded from 64 scalp sites using electrodes mounted in an elastic cap (NeuroScan, USA). Electrode impedance was kept below 10 kΩ, and the data were sampled at 1000 Hz. The online reference electrode was positioned between CPz and Cz. Horizontal electrooculogram (HEOG) was recorded via two electrodes placed 1.5 cm lateral to each eye, and vertical electrooculogram (VEOG) was recorded using electrodes placed above and below the left eye.

Data preprocessing was conducted using EEGLAB [35] and ERPLAB [36]. A notch filter at 50 Hz and a bandpass filter from 0.05 to 40 Hz were applied. The EEG data were re-referenced to the average of all scalp channels. Independent component analysis was used to remove artifact components related to eye movements. The continuous data were segmented into epochs ranging from − 200 ms to 600 ms relative to the onset of morally relevant pictures. Baseline correction was performed using the pre-stimulus interval from − 200 to 0 ms. To ensure data quality, trials meeting any of the following criteria were excluded from analysis. First, epochs immediately after participants’ responses were excluded to eliminate contamination from keystroke-related activity. Second, the epochs with activity exceeding ± 35 µV on the VEOG, ± 25 µV on the HEOG, or ± 75 µV on other channels were excluded to avoid potential artifacts. On average, there were 440 ± 6/110 ± 3 (*M* ± *SD*) trials for standards/deviants in the moral condition and 438 ± 16/109 ± 5 trials for standards/deviants in the immoral condition.

## Statistical analysis

### Behavioral data

Using SPSS 22.0 software, paired samples *t*-tests were conducted to assess the accuracy in both phases A and B.

### Event-related potentials

The difference wave is obtained by subtracting the ERPs to the standards from the ERPs to the deviants. To keep the stimuli physically identical, the vMMR was obtained as the difference of ERPs between standards in the oddball sequence and the deviants in the reversed block (or vice versa) [15, 33]. Thus, there are two difference waves of moral (deviant moral vs. standard moral) and immoral conditions (deviant immoral vs. standard immoral). The vMMR is typically characterized by a differential response between deviant and standard stimuli at posterior electrodes around 150–400 ms post-stimulus [11]. In the present study, the grand-averaged waveforms revealed distinct vMMR components at electrodes PO7 and PO8 within the parieto-occipital region, specifically during two time windows: 100–250 ms and 300–400 ms. Moreover, previous studies [15, 37] have analyzed vMMR using single or a limited number of electrodes, supporting the feasibility of this approach in specific research contexts. Accordingly, we selected PO7 and PO8 electrodes and the 100–250 ms and 300–400 ms time windows for our analysis. The data from phase A and phase B were analyzed collectively. A four-factor repeated-measures ANOVA was performed in two time windows of 100–250 ms and 300–400 ms, respectively. The factors included task relevance (task-irrelevant, task-relevant), moral valence (moral, immoral), stimulus type (standard, deviant), and hemisphere (PO7, PO8). The *P*-values were corrected using the Greenhouse-Geisser method when violations of the spherical hypothesis occurred. To account for multiple comparisons, a Bonferroni correction was applied. Partial eta squared is used to measure the effect size of an independent variable on a dependent variable [38]. The interpretation criteria are as follows: 0.01 indicates a small effect, 0.06 indicates a medium effect, and 0.14 indicates a large effect [39].

## Results

### Post-test ratings

The post-test ratings exhibited similar scoring patterns as the pre-test ratings. Repeated-measures ANOVA with picture type (moral, immoral, and neutral) was performed separately for each dimension (see Table 3). The main results were as follows: (1) Moral content: The main effect of picture type was significant [*F*(2, 38) = 252.89, *P* < 0.001, $\:{\eta\:}_{p}^{2}\:$= 0.93], the moral content of moral pictures was higher than that of immoral (*P* < 0.001) and neutral pictures (*P* < 0.001), and the moral content of neutral pictures was higher than that of immoral pictures (*P* < 0.001); (2) Emotional arousal: The main effect of picture type was significant [*F*(2, 38) = 13.98, *P* < 0.001, $\:{\eta\:}_{p}^{2}\:$= 0.42]. There was no difference in emotional arousal between moral and immoral pictures (*P* = 0.123), whereas neutral pictures elicited lower arousal compared to moral (*P* = 0.002) and immoral pictures (*P* < 0.001); (3) Harm: The main effect of picture type was significant [*F*(2, 38) = 125.22, *P* < 0.001, $\:{\eta\:}_{p}^{2}\:$= 0.87]. The harm of moral pictures was lower than that of immoral pictures (*P* < 0.001) and neutral pictures (*P* = 0.01), and the harm of neutral pictures was lower than that of immoral pictures (*P* < 0.001).

**Table 3:** Post-test ratings of moral content, emotional arousal, and harm of stimuli [*M* (*SE*)]

	Picture type		
	Moral	Immoral	Neutral
Moral	8.23 (0.14)	2.02 (0.16)	5.88 (0.28)
Emotional arousal	6.45 (0.22)	6.79 (0.22)	5.28 (0.36)
Harm	1.48 (0.14)	7.15 (0.19)	2.53 (0.40)

### Behavioral data

The accuracy (see Table 4) of phase B was found to be higher than that of phase A in a paired-samples *t*-test [*t*(19) = 3.83, *P* = 0.001, *d* = 0.98]. This could be attributed to the differences in the experimental tasks between the two phases. Notably, in this study, selective attention to morally relevant stimuli was manipulated through task relevance. During phases A and B, the participants were unaware that some of the images were morally relevant stimuli and were not required to make moral evaluations of the images. In phase A, the participants were tasked with judging the change of the central fixation point, making the morally relevant stimuli task-irrelevant in this phase. The high task accuracy rate of phase A (0.91) indicated that participants primarily focused on the fixation point, leaving the task-irrelevant morally relevant stimuli unattended. In phase B, the participants were tasked with judging the appearance of the target images, rendering the morally relevant stimuli in this phase task-relevant. The task accuracy rate for phase B was 0.96, suggesting that participants allocated their attention resources to the pictures, demonstrating sustained attention to the morally relevant stimuli during the experiment.

**Table 4:** Reaction time (ms), accuracy, and the number of false alarm responses of two experimental phases [*M* (*SE*)]

Phase	RT	Accuracy	the number of false alarm response
A	687 (15)	0.91 (0.01)	0.25 (0.14)
B	610 (13)	0.96 (0.01)	0.15 (0.08)

### Event-related potentials

**Fig. 2:**
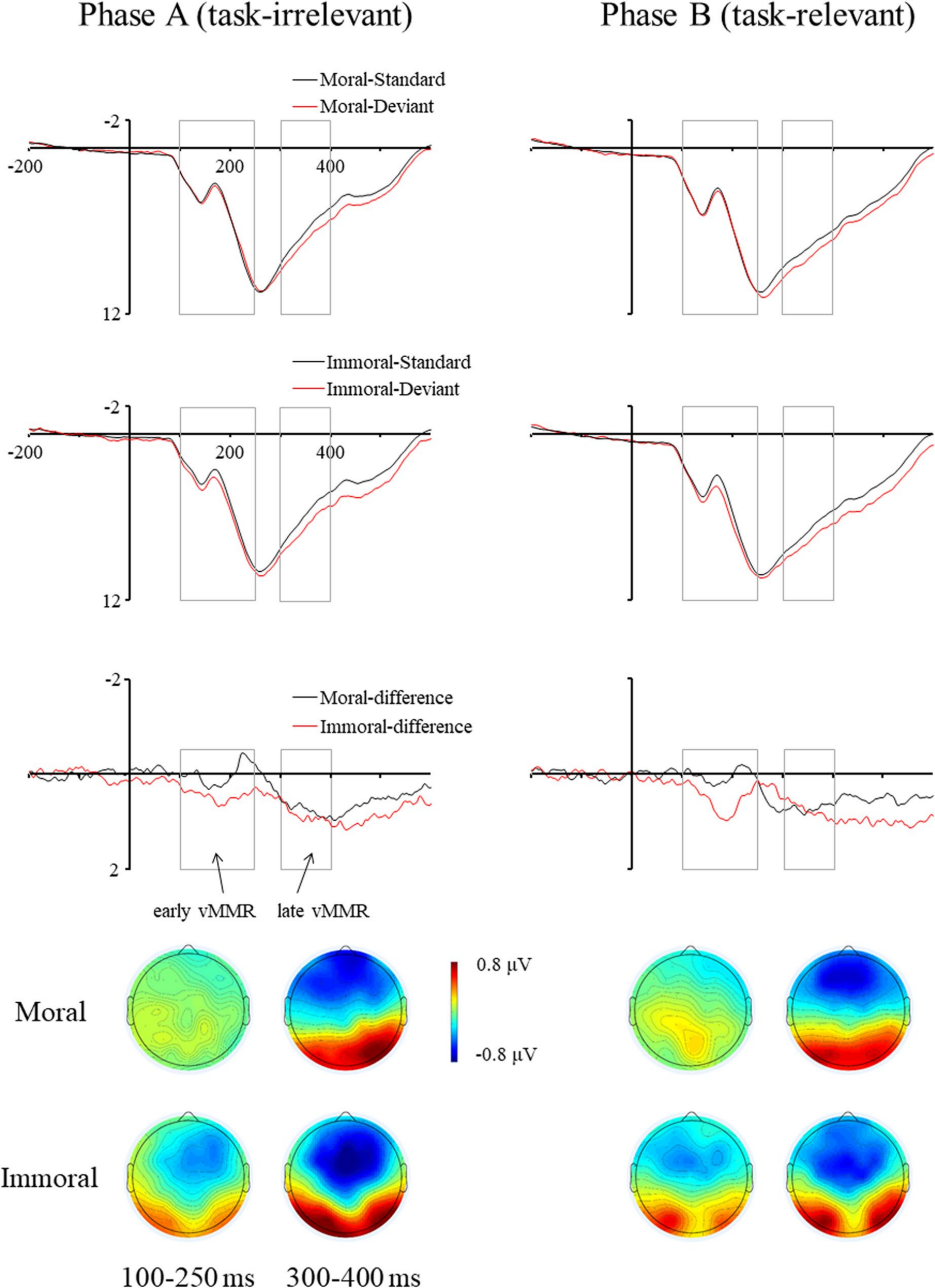
Grand-averaged ERPs elicited by standards and deviants under task-irrelevant and task-relevant conditions (first row is for moral stimuli, second row is for immoral stimuli), difference waves, and scalp topographies of the difference waves. The gray rectangles in the third row indicate the early 100-250ms and late 300-400ms vMMR respectively

The data from phase A and phase B were analyzed together (see Fig. 2). A four-factor repeated-measures ANOVA was performed in two time windows of 100–250 ms and 300–400 ms, respectively. The factors included task relevance (task-irrelevant, task-relevant), moral valence (moral, immoral), stimulus type (standard, deviant), and hemisphere (left, right). The main results are as follows.

The 100–250 ms time window results showed a significant main effect of stimulus type [*F*(1, 19) = 13.59, *P* = 0.002, $\:{\eta\:}_{p}^{2}\:$= 0.42], and the amplitude of deviants was significantly more positive than that of standards [deviant = 5.20 ± 0.49 µV, standard = 4.96 ± 0.49 µV]. The interaction between stimulus type and moral valence was significant [*F* (1, 19) = 13.50, *P* = 0.002, $\:{\eta\:}_{p}^{2}\:$= 0.42]. Simple effect analysis showed that there was no difference between standards and deviants in the moral condition [deviant = 5.05 ± 0.48 µV, standard = 5.05 ± 0.49 µV, *P* = 0.995], but the amplitude of the deviants was significantly more positive than that of the standards in the immoral condition [deviant = 5.35 ± 0.50 µV, standard = 4.87 ± 0.48 µV, *P* < 0.001]. This showed that immoral stimuli induced early vMMR, whereas moral stimuli failed to evoke early vMMR, indicating the automatic processing of moral information induced early vMMR with a negative bias. Moreover, the interaction between stimulus type and task relevance, as well as the interaction of both with other factors, were not significant (*Ps* > 0.693). This suggested that the amplitude of the early vMMR was unaffected by task relevance.

The 300–400 ms time window results showed a significant main effect of stimulus type [*F*(1, 19) = 36.66, *P* < 0.001, $\:{\eta\:}_{p}^{2}\:$= 0.66], the amplitude of the deviants was significantly more positive than that of the standards [deviant = 7.27 ± 0.70 µV, standard = 6.51 ± 0.62 µV]. This suggested that the automatic processing of both moral and immoral stimuli induced late vMMR. In addition, the interaction of stimulus type with task relevance and the interaction of both with other factors were not significant (*Ps* > 0.255). This suggested that the amplitude of late vMMR was not affected by task relevance.

## Discussion

This study investigated the automatic processing of moral information by using the vMMR as an index of automatic change detection. The main findings are as follows. First, an early vMMR was elicited selectively by immoral stimuli when they appeared as deviants; no such effect was observed for moral deviants. This finding carries two implications: it is consistent with the existence of an automatic neural process in the early stages of moral judgment (aligning with the concept of moral intuition), and it further reveals a negativity bias within this automatic detection mechanism. Second, the amplitude of this early vMMR was not modulated by task relevance, suggesting that this neural detection operates independently of directed attention. Finally, both moral and immoral stimuli elicited a late vMMR. The presence of both early and late components suggests that automatic moral processing unfolds across multiple temporal stages, encompassing both rapid deviance detection and subsequent, more in-depth automatic analysis.

### Rapid automatic processing of moral information

Although the social-intuitionist model suggests that moral judgment is determined by rapid and automatic moral intuition [1], the extent of the automatic processing of moral intuition remains unclear. Tao et al. [9] used an implicit moral task to examine the time course of moral judgment but their paradigm could not fully dissociate automatic processing from the effects of task-driven attention or low-level stimulus features. Therefore, the current study used vMMR—an electrophysiological index of automatic change detection—to explore the automatic processing of moral information under conditions where morally relevant pictures were task-irrelevant and physical stimulus differences were controlled via a reverse-block design. The results showed that immoral stimuli, when presented as deviants, elicited an early positive vMMR. Because vMMR reflects pre-attentive regularity extraction and prediction violation, this finding suggests that at least some aspects of moral content—particularly immoral information—are processed automatically. This aligns with proposals that early neural components such as the N1 [2] and N2 [9] may index moral intuition. Thus, the presence of an early vMMR in response to immoral deviants provides converging evidence that moral information can be detected automatically in the early stages of processing, consistent with the notion of rapid moral intuition.

Early vMMR is not equated directly with moral intuition; instead, it is regarded as an index reflecting the brain’s automatic detection of deviations in moral content—a process interpreted as the operation of moral intuition under non-attentive conditions. This interpretation is grounded in predictive coding theory: if the brain can extract regularities from repeatedly presented moral stimuli and generate prediction error signals when those regularities are violated, it suggests that moral categories are being processed automatically and pre-attentively—an essential feature of moral intuition as proposed by the social intuitionist model [1]. Although early vMMR fundamentally reflects automatic change detection, in the context of our study, moral information serves as task-irrelevant background stimuli, and the early vMMR elicited by its automatic processing can be seen as a manifestation of moral intuition. Previous research has demonstrated that vMMR can be used to probe the automatic processing of socially significant information, such as emotional [21] and moral content [37].

It should be noted that although the early vMMR finding is consistent with the notion of moral intuition, it may also be more conservatively interpreted as reflecting a sensitivity to changes in moral categories, rather than constituting direct evidence for moral intuition per se. In other words, the early vMMR could be understood as the automatic detection of a moral-category shift. In the present paradigm, the oddball sequences established a categorical context of moral versus immoral, and the early vMMR could reflect the brain’s rapid identification that the current stimulus belongs to a moral category that violates the preceding categorical prediction.

### Early vMMR is unaffected by task relevance

The present study manipulated selective attention to morally relevant pictures through task relevance. Specifically, the pictures were unattended under the task-irrelevant condition (Phase A), whereas participants selectively attended to the pictures under the task-relevant condition (Phase B), though the moral content itself remained task-irrelevant in both phases. Notably, even in the task-relevant condition where participants responded to neutral target pictures rather than moral information, vMMR continued to reflect automatic processing of moral deviance. This paradigm is consistent with previous studies that have explored automatic processing of facial emotion under conditions where faces are task-relevant but emotion is task-irrelevant [40, 41], as well as studies where both faces and emotion are task-irrelevant [15, 20]. The results showed that the amplitude of early vMMR was not modulated by task relevance. This finding suggests that the neural mechanism underlying automatic detection of moral deviance operates independently of directed attention, which is consistent with the view that moral intuition involves highly automatic processes. In line with this, Schlossmacher et al. [42] reported that task relevance does not affect the automatic processing of basic visual features.

The absence of task relevance effects on early vMMR warrants further consideration in light of predictive coding accounts [10, 27]. From this perspective, vMMR reflects perceptual prediction error signals, and attention is proposed to enhance the precision of predictions by modulating the synaptic gain of prediction error units. According to this view, violations of more precise predictions should elicit larger prediction error signals. Contrary to this prediction, the present study found that manipulating attention via task relevance did not modulate early vMMR amplitude. One possible interpretation is that individuals extracted regularities from the moral stimulus environment and formed predictions with comparable precision under both task-relevant and task-irrelevant conditions. This may be attributable to the perceptual salience of moral information. For instance, previous findings suggest that morally relevant words capture attention more quickly [22, 25] and gain prioritized access to visual consciousness [23] compared to neutral words. Thus, the perceptual advantage of moral information might facilitate rapid visual processing independent of task-directed attention, potentially explaining why early vMMR was unaffected by task relevance.

### Early vMMR reveals a negativity bias in automatic moral processing

The present study found that early vMMR was elicited only by immoral stimuli when they appeared as deviants, whereas moral deviants did not evoke such a response. This pattern suggests a negativity bias in the early, automatic detection of moral information. In line with this, Tao et al. [9] reported that immoral pictures elicited larger N2 amplitudes than moral pictures, also pointing to differential processing of moral and immoral content at early stages. One possible explanation for this negativity bias is that immoral stimuli, as negative emotional stimuli, may rapidly capture attention. Previous research has consistently demonstrated the strong attention-capture effect of negative emotional stimuli [43, 44]. The rapid processing of threatening negative emotional information aids individuals in assessing their surrounding environment and responding with appropriate behaviors [45], which is crucial for survival [46]. However, it should be noted that findings on early bias are not entirely consistent; for instance, Yoder and Decety [6] proposed a positive bias in early moral judgment, as reflected by higher N1 and N2 amplitudes for moral compared to immoral stimuli. Consequently, further investigation is necessary to verify the presence of a negative bias in automatic moral processing.

The negativity bias observed in the present study can be interpreted within the neurocognitive model proposed by Reynolds [47], which links moral intuition to automatic, unconscious processing. According to this model, when individuals encounter morally relevant stimuli, the brain employs pattern-matching principles to identify new stimuli, with stored moral prototypes automatically extracted from memory. Common moral transgressions—such as bribery, theft, lying, and sexual harassment—may serve as salient prototype features stored in long-term memory. This pattern-matching process enables rapid and automatic recognition of immoral situations and behavioral norms. Thus, the storage of immoral behavior prototypes, combined with automatic pattern-matching mechanisms, may contribute to the rapid identification of immoral stimuli observed in the present study, offering a plausible mechanism for the automatic elicitation of early vMMR by immoral deviants.

Although the present study found that immoral stimuli elicited early vMMR, which may suggest a negative bias in the automatic processing of moral information, this interpretation should be approached with caution. Immoral scenes often contain perceptually salient features (injury, conflict, blood) that could enhance prediction error signals due to low-level visual characteristics. Moreover, negative emotional stimuli are generally processed more rapidly across various cognitive domains [23, 44, 48]. Therefore, the observed effect may partly reflect general emotional salience or threat detection mechanisms rather than being specific to moral negativity. Future studies should include morally neutral affective stimuli matched on valence and arousal to better disentangle the contribution of moral content from emotional salience in early vMMR.

Previous research suggests that there may be a perceptual advantage for moral information, indicating that it can capture attention more quickly and gain preferential access to consciousness [22, 23, 25]. The present study seeks to further explore this by focusing on moral valence and examining the automatic processing of moral information, using vMMR as an indicator. The findings reveal a negativity bias underlying this moral perceptual advantage, suggesting that even at the pre-attentive stage, the cognitive system tends to give priority to negative moral information. This result aligns with research on negativity bias in the domain of emotional processing [44, 48]. For instance, Huang et al. [44] found that negative emotional stimuli can automatically capture attention during the early stages of visual search. Taken together, it appears that negativity bias is not unique to emotional information but is also present in the early processing of moral information, which may reflect individuals’ heightened sensitivity to socially threatening stimuli.

### Early and late vMMR suggest multi-stage automatic processing of moral information

The late vMMR in the present study was derived from differences in the P3-related late component. Although previous studies have suggested that the late P3 component may be associated with cognitive evaluation of moral information [2, 9] and is often considered to reflect higher-order cognitive processes [49], within the automatic processing paradigm employed here, the early and late vMMR may be more coherently interpreted as neural signatures of multi-level automatic processing of moral information. Specifically, the early vMMR may reflect rapid, initial automatic detection of deviance in moral information, while the late vMMR may represent subsequent, deeper or more fine-grained automatic analysis of the deviant information. This interpretation is compatible with the synergistic processing flow described by the cognitive-intuitionist model [4], wherein an intuitive response is followed by further automatic cognitive processing.

This interpretation aligns with Reynolds’ [47] proposal of a synergistic relationship between the X-system and C-system. The X-system is responsible for rapid, unconscious pattern matching, while the C-system can be recruited for more in-depth analysis when necessary. In the context of the present study, it is plausible that the activity of both systems may manifest as automatic processing: the X-system performs initial rapid detection of deviance in moral stimuli, eliciting the early vMMR; subsequently, the C-system may be automatically engaged to conduct a more thorough analysis or confirmation of the same deviant moral information, with this automatic depth-processing potentially reflected in the late vMMR. When a deviant stimulus appears, the system first rapidly flags the anomaly (early vMMR), and then automatically initiates deeper-level evaluation to consolidate or refine the encoding of this deviance (late vMMR), together forming a coherent sequence of automatic processing.

The results show that automatic moral processing sequentially elicits early and late vMMR. Whereas the late vMMR correlates with the late P3 component and involves fine-grained processing [2, 49], under automatic processing it more parsimoniously reflects in-depth automatic analysis of previously detected moral information, rather than a shift to conscious, controlled moral reasoning.

Theoretical debates surrounding moral judgment often center on the dichotomy between intuition and reasoning [1, 3]. The present findings offer a complementary perspective for understanding this process: the processing of moral information may involve a multi-level automatic system. Within this system, initial rapid detection (associated with intuition) and subsequent deep automatic analysis (which may encompass both intuitive and automatic cognitive features) operate synergistically. Therefore, the results of this study do not fully support a strict dichotomy between intuition and reasoning, but rather align more closely with the spirit of continuity and synergistic processing emphasized in the cognitive-intuitionist model [4]. Within this framework, the early vMMR may mark the rapid capture of stimulus deviance by processes associated with moral intuition, while the late vMMR may represent automatic depth-processing of moral information by the cognitive system building upon the preceding stage. Together, they may contribute to effective processing of moral information under implicit conditions.

Recently, Sun et al. [37] employed vMMR as an indicator to explore how emotional arousal influences the automatic processing of moral information. The findings suggest that both emotional and moral information can be automatically processed at an early stage, and that emotion may play a role in later stages of moral processing. Building upon this foundation, the present study examines the automatic processing of moral information under both task-relevant and task-irrelevant conditions. The findings of the present study offer insight into a potential negativity bias in the early processing of moral information and suggest that task relevance does not significantly modulate the automatic detection of such information. Together, these two studies provide complementary perspectives that enhance our understanding of the multi-stage automatic processing of moral information.

### Limitations and prospects

There are some limitations to this study. First, the absence of a neutral control condition within the oddball sequence limits our ability to distinguish morally specific processing from general visual deviation detection. Future research should incorporate neutral pictures as both standard and deviant stimuli, enabling direct comparisons to determine whether moral content elicits prediction error signals distinct from those triggered by morally neutral perceptual changes.

Second, the relationship between emotion and moral judgment remains unclear. Building on the valuable insights from Sun et al. [37], who used vMMR to track the temporal divergence of emotional and moral processing, and from Cenka et al. [28], who revealed dynamic transitions from empathy to mentalizing, a promising avenue for future research would be to further clarify the neural sources underlying these temporal dynamics. Given the inherent spatial limitations of EEG, integrating vMMR with fMRI could provide complementary information about where these processes occur in the brain. Such multimodal approaches might help explore whether early automatic detection of immoral content (early vMMR) involves regions such as the amygdala or superior temporal sulcus [50, 51], and whether later emotion-modulated processing (late vMMR) relates to engagement of mentalizing or cognitive control networks, as suggested by previous fMRI studies [52–54]. This line of inquiry could potentially deepen our understanding of not only that emotion and cognition interact, but also how and where this interaction unfolds.

Third, the vMMR effects may be associated with valence, threat, or low-level visual features. In the present study, we attempted to control for arousal and physical differences. By employing a reverse block design, we sought to minimize low-level visual confounds, which helped ensure that the vMMR reflects, at least in part, the processing of moral content rather than mere changes in physical features. Moreover, the early vMMR appeared significant only in the immoral condition, but not in the moral condition, which might suggest a degree of moral specificity beyond general deviance detection. Nevertheless, future studies could further dissociate moral content from other dimensions by comparing moral and non‑moral affective stimuli matched on valence and arousal. The semantic complexity of immoral scenes might also play a role in facilitating deviance detection. Thus, while the findings are broadly consistent with the notion of automatic moral intuition, alternative interpretations cannot be entirely excluded. Combining vMMR with more direct moral measures may help further clarify the unique contribution of moral content to neural processing.

Fourth, moral judgment is inherently multidimensional, encompassing various moral foundations such as care/harm, fairness/cheating, loyalty/betrayal, authority/subversion, sanctity/degradation, and liberty/oppression [29, 30]. The present study focused exclusively on the harm/care dimension, which may not fully capture the complexity of moral cognition. Different moral foundations may engage distinct psychological and neural processes [31], and the automatic processing of moral information may vary across domains. Therefore, future studies should include stimuli representing a broader range of moral dimensions to examine whether the automaticity and temporal dynamics observed here are domain-general or dimension-specific. Such efforts would help build a more comprehensive model of implicit moral processing.

Fifth, given that moral judgments are shaped by culture, drawing general conclusions from a culturally homogeneous participant group inherently limits the applicability of the findings. In particular, the stimulus materials used in this study predominantly depict scenes from Western social contexts, whereas the participants were all drawn from a Chinese cultural background. Although post-test ratings confirmed that these Chinese participants also evaluated the immoral pictures as morally unacceptable, we cannot assume that participants from all cultural backgrounds would evaluate the same stimuli in an identical manner. There is likely an interaction among participants’ moral beliefs, the sociocultural representations embedded in the stimulus materials, and their own cultural context. For example, behaviors considered prototypically immoral in Western cultures might be assigned different moral weight or provoke different intensities of moral intuition in Chinese culture. Therefore, when interpreting the automatic moral processing effects observed here, it is currently more appropriate to restrict the conclusions to outcomes observed within a Chinese cultural sample. Future studies should systematically adopt cross-cultural paradigms, directly comparing vMMR effects elicited by the same moral stimuli across different cultural groups to test whether the observed neural patterns are universal or culturally modulated. Such comparisons would help assess the ecological validity of the present findings and deepen our understanding of how culture shapes moral cognition.

Sixth, this study lacked a systematic post-experimental awareness check, so we cannot entirely rule out that some participants incidentally noticed the moral content, which may confound the interpretation of implicit processing. Future studies should employ structured interviews or recognition tasks to quantify awareness and better dissociate implicit from explicit moral processes.

Seventh, the vMMR indexes the brain’s automatic detection of deviations from established regularities—a form of pre-attentive change detection. While this detection process may constitute an early stage of moral intuition, it is not equivalent to moral judgment, which typically involves evaluative processes that assign value (e.g., right/wrong, good/bad) to actions or agents. Our findings demonstrate that the brain can automatically detect morally relevant information—particularly negative moral content—without conscious attention, but they do not directly demonstrate that such detection constitutes a moral judgment. Future research employing paradigms that can dissociate detection from evaluation will be necessary to establish the link between automatic moral information detection and implicit moral judgment.

Eighth, given that the present design did not independently manipulate moral relevance and emotional valence, we cannot rule out the possibility that the observed early vMMR reflects a broader negativity detection mechanism rather than a morality-specific process. The negativity bias observed here may reflect the brain’s general sensitivity to potentially threatening or harmful stimuli, of which immoral acts constitute one important subcategory. To isolate the moral component of this effect, future studies could employ a fully crossed design in which moral/immoral and negative/positive emotional stimuli are orthogonally manipulated.

## Conclusion

This study employed the vMMR—an index of automatic change detection—to investigate the temporal dynamics of automatic moral information processing. The results revealed that an early vMMR was elicited only by immoral pictures when they appeared as deviants, and critically, the amplitude of this effect was not modulated by task relevance. This pattern aligns with the notion of a fast and highly automatic moral intuition. Furthermore, a late vMMR was evoked by both moral and immoral pictures. The co-occurrence of early and late vMMR components suggests that the automatic processing of moral information is not a monolithic process but unfolds across multiple temporal stages, encompassing rapid deviance detection followed by more in-depth automatic analysis.

## Data Availability

The datasets used and/or analyzed during the current study are available from the corresponding author on reasonable request.

## Abbreviations

ANOVA: Analysis of variance
EEG: Electroencephalography
ERP: Event-related potential
fMRI: Functional magnetic resonance imaging
HEOG: Horizontal electrooculogram
MFT: Moral Foundations Theory
SMID: Socio-Moral Image Database
vMMR: Visual mismatch response
VEOG: Vertical electrooculogram
